# Evaluation of Two Compressed Air Foam Systems for Culling Caged Layer Hens

**DOI:** 10.3390/ani8050061

**Published:** 2018-04-24

**Authors:** Eric R. Benson, Jaclyn A. Weiher, Robert L. Alphin, Morgan Farnell, Daniel P. Hougentogler

**Affiliations:** 1 Department of Animal and Food Sciences, University of Delaware, Newark, DE 19716, USA; jweiher@udel.edu (J.A.W.); ralphin@udel.edu (R.L.A.); dhoug@udel.edu (D.P.H.); 2 Department of Poultry Science, Texas A&M University, College Station, TX 77843, USA; mfarnell@tamu.edu

**Keywords:** depopulation, foam, cage, layer hen, chicken, poultry, emergency, euthanasia, culling methods

## Abstract

**Simple Summary:**

Control of avian influenza and similar diseases in commercial poultry operations is challenging; the six major steps are surveillance, biosecurity, quarantine, depopulation, disposal, and cleaning and disinfection. Depopulation is used to cull animals that are terminally ill and to reduce the number of animals that can spread an untreatable disease. Water-based foam depopulation was used effectively during the 2014–2015 highly pathogenic avian influenza outbreak in the United States. Water-based foam, however, cannot be used effectively in caged poultry operations. Compressed air foam systems were initially developed for structural fire-fighting and, with modifications, can provide the conditions required to effectively penetrate a poultry cage and provide sufficient residence time for depopulation. In this experiment, compressed air foam was used to depopulate caged layer hens. Compressed air foam resulted in faster unconsciousness than carbon dioxide gassing. The experiment demonstrated that compressed air foam systems have promise for depopulating birds raised in cages.

**Abstract:**

Outbreaks of avian influenza (AI) and other highly contagious poultry diseases continue to be a concern for those involved in the poultry industry. In the situation of an outbreak, emergency depopulation of the birds involved is necessary. In this project, two compressed air foam systems (CAFS) were evaluated for mass emergency depopulation of layer hens in a manure belt equipped cage system. In both experiments, a randomized block design was used with multiple commercial layer hens treated with one of three randomly selected depopulation methods: CAFS, CAFS with CO_2_ gas, and CO_2_ gas. In Experiment 1, a Rowe manufactured CAFS was used, a selection of birds were instrumented, and the time to unconsciousness, brain death, altered terminal cardiac activity and motion cessation were recorded. CAFS with and without CO_2_ was faster to unconsciousness, however, the other parameters were not statistically significant. In Experiment 2, a custom Hale based CAFS was used to evaluate the impact of bird age, a selection of birds were instrumented, and the time to motion cessation was recorded. The difference in time to cessation of movement between pullets and spent hens using CAFS was not statistically significant. Both CAFS depopulate caged layers, however, there was no benefit to including CO_2_.

## 1. Introduction

Avian diseases such as exotic Newcastle disease, highly pathogenic avian influenza (HPAI), and others continue to be a concern for the poultry industry. Recent events have highlighted the potential danger to the commercial poultry industry, with the North American outbreaks of 2014–2015 and 2016 as significant events. In November 2014, an outbreak of H5N2 HPAIV was detected by broiler breeders in Fraser Valley, Canada. The outbreak expanded into the United States, and by June 2015, the H5N2 and H5N8 viruses had been detected on 236 premises in 15 states [1,2,3]. The outbreak resulted in the death or depopulation of 7.5 million turkeys and 42.1 million egg-layer and pullet chickens. In the 2014–2015 U.S. outbreak, wild bird movements were the likely source of the initial introduction and subsequent spread of HPAI into new areas [2]. However, once HPAI was in a region, poor biosecurity and human activity became an important transmission mechanism. The outbreak resulted in a total economic loss of approximately $3.3 billion, with outbreak control estimated at $850 million [1,3]. In contrast, the 2016 H7N8 HPAIV outbreak was more quickly contained and resulted in one confirmed turkey premise, one dangerous contact layer chicken premise, and nine LPAI-affected turkey premises [3].

The approach for dealing with such contagious diseases includes surveillance, biosecurity, quarantine, depopulation, disposal, and decontamination. Depopulation of the diseased flock minimizes animal suffering and stops viral replication and dissemination. The American Veterinary Medical Association (AVMA) has outlined the animal welfare standards for both general euthanasia and depopulation during outbreaks. Euthanasia methods for poultry (domesticated birds used for egg, meat, or feather production, e.g., chickens, turkeys, quail, pheasants, ducks, geese), include gas inhalation, manually applied blunt force trauma, cervical dislocation, decapitation, electrocution, gunshot, captive bolt, and injectable agents [4]. Water-based foam is considered as ‘allowed in constrained circumstances’ for depopulation and causes occlusion of the trachea, preventing oxygen from reaching the lungs [5]. 

The AVMA supports the use of water-based foam as a method of mass depopulation in accordance with the conditions and performance standards outlined by the US Department of Agriculture’s Animal and Plant Health Inspection Service (USDA APHIS) [5]. The conditions are as follows: (1) Appropriate method of depopulation for floor-reared poultry, (2) animals are potentially infected with a zoonotic disease, (3) animals are experiencing an outbreak of a rapidly spreading infectious disease that, in the opinion of state or federal regulatory officials, cannot be contained by conventional or currently accepted means of depopulation, and (4) animals are housed in structurally unsound buildings that would be hazardous for human entry, such as those that may result from natural disaster [5].

The two methods of water-based foam depopulation currently available are foam generator systems and air aspirating nozzle based systems. Foam can be defined as a stable aggregation of water and foam concentrate that has been expanded by air or other gas to create a bubble structure [6]. A pump, typically gasoline or diesel powered, provides energy to the system. The surfactant based foam concentrate can be batch mixed, educted, or proportioned to match water flow to the desired foam concentration. With an air aspirating nozzle, air is entrained into the foam drawing air into a stream of surfactant–water solution inside the nozzle [6,7]. With a foam generator, the water flow from the pump drives a water powered motor that is directly connected to a fan to aerate the mixture.

Gassing procedures are one of the primary alternatives for mass emergency depopulation. Gassing can be implemented as containerized gassing, partial house gassing, or whole-house gassing [8,9,10]. In containerized gassing, individual birds are manually placed into a chamber that is charged with an anoxic gas [8,9]. Partial house gassing requires reducing the size of the facility by confining birds to a portion of the facility and adding temporary sealing to limit the size and scope of the area treated with gas [9]. Whole-house gassing requires sealing of all openings of the house and rapidly vaporizing large quantities of gas into the house, which can be performed while requiring minimal handling of live birds [10]. Argon (Ar), nitrogen (N_2_), carbon dioxide (CO_2_), or mixtures of these gases are most commonly recommended for depopulation [11]. With gassing procedures, speed of oxygen displacement and loss of consciousness should be balanced against awareness to the process. Carbon dioxide is a well-known anesthetic gas that can induce rapid loss of consciousness, however, at high concentrations (>65%), it is known to be an irritant to humans [12,13]. Argon and nitrogen are examples of inert gases that are difficult for birds to detect; these gases displace oxygen resulting in hypoxia or anoxia [14].

Dry foam is an alternate foam depopulation method in which a high expansion (300:1) foam is used as the delivery mechanism for an anoxic gas (i.e., CO_2_ or N_2_). Wet foams act directly to block oxygen from reaching the lungs, while dry foams create a localized, oxygen deficient environment, based on release of the included gas [15]. In initial testing, dry foam was able to rapidly cause unconsciousness in multiple species during individual trials.

Compressed air foam systems (CAFS) are designed for fire suppression, and utilize a homogenous foam that includes a combination of water, foam concentrate, air, N_2_, or other gases under pressure [16]. In CAFS, air is injected from a high pressure cylinder or air compressor prior to distribution which results in a foam with different characteristics than a foam generator, air aspirating nozzle, or dry foam systems. CAFS are much more effective in suppressing compartment fires than with water alone or with a foam–water solution [6]. CAFS also have a higher retention time than the other types of foam tested [7], which provides a higher retention time in objects such as animal cages. Gurung et al. [17] found that CAFS with N_2_ resulted in faster time to motion cessation. Gurung et al. [18] found that CAFS with and without inert gas resulted in similar bird stress levels.

An outbreak of HPAI in caged layers is one of the worst-case scenarios for the current poultry industry. Cages found in layer houses present significant difficulty for depopulation because the birds either need to be removed prior to depopulation or immediately after depopulation, significantly increasing the labor and time requirements. Depopulating birds in cages impacts which methods are most appropriate. Nozzle based and generator based foam depopulation systems can effectively kill layer hens outside of cages, however, the foam cannot effectively penetrate and remain within a cage system. Currently there are no foam depopulation methods that are effective, fast enough, and suitable for large caged layer operations. 

The objective of this study was to evaluate different methods of depopulation for layer hens using a novel type of foam, compressed air foam, and CO_2_ gas. 

## 2. Materials and Methods 

Experiment 1 evaluated the suitability of CAFS for depopulating 176 spent layer hens in commercial cages with multiple birds per cage. One of three randomly selected depopulation methods: CAFS, CAFS with CO_2_ gas, and CO_2_ gas were randomly assigned to each trial. The time to cessation of movement (COM), unconsciousness, brain death, and altered terminal cardiac activity (ATCA) were recorded. 

Experiment 2 used a different CAFS system, with bird age (pullet or spent hens) as a variable for the 144 birds in the trial. A single treatment with CAFS was used. The time to COM was recorded.

For Experiments 1 and 2, trials were conducted in a 21.3 m × 9.1 m layer facility at the University of Delaware Agricultural Experiment Station in Newark, Delaware. The facility was equipped with 240, 0.4 m × 0.47 m × 0.3 m commercial layer cages, with four hens per cage representing standard commercial stocking density for a total capacity of 960 hens. There were two parallel rows of layer cages, two levels high, equipped with manure belts.

### 2.1. Experiment 1

CAFS was tested using a randomized block design with commercial layer hens treated with one of three randomly selected depopulation methods: CAFS, CAFS with CO_2_ gas, and CO_2_ gas. Partial house CO_2_ gassing was used as a control treatment. A total of 176 spent layer hens (birds > one year of age) were used for the study. Of the 176 birds, 44 were surgically instrumented with a wireless Electroencephalography (EEG) transmitter (Data Sciences International, St. Paul, MN, USA). The surgically instrumented birds were also outfitted with ECG electrodes and an accelerometer. A total of three data sets were collected for each of the 44 surgically instrumented birds, comprising a total of 132 readings collected. On the following day after surgery, the four surgically instrumented birds underwent a randomly selected treatment (CO_2_ gas, CAFS, or CAFS w/CO_2_ gas). For each surgically instrumented bird there was also a companion bird located in the same cage, and two additional birds located in an adjacent cage. These three birds were instrumented with accelerometers and data was collected from each, resulting in an additional 132 data sets being collected. Rubber chickens were placed in the cages surrounding the birds to simulate how foam would flow around birds in the surrounding cages without having to increase the number of animals used in this study. All birds were housed in one row of layer cages of the facility and moved to the other row of layer cages for the depopulation trial.

A 180 s (three minute) baseline period was recorded to establish normal ECG and EEG patterns. After the baseline was recorded, there was a 900 s (15 min) treatment period with a 180 s application time included. Data were collected from all three sensors for the duration of the trial. The data collected were analyzed to find their respective critical physiological points. The time to unconsciousness and brain death were determined using raw and frequency domain EEG data, time to cessation of movement (COM) was determined from the accelerometer data, and time to altered terminal cardiac activity (ATCA) was determined from ECG data. All times were reported from the beginning of gas application or submersion in foam. All testing was performed under the approval and guidelines of the University of Delaware Agricultural Animal Care and Use Committee (Protocol (33) 02-24-14R, 7 Revised 6 March 2014 and followed the guidelines laid out by the Federation of Animal Science Societies [19].

### 2.2. Experiment 2

CAFSs were tested with commercial layer hens with a single CAFS methodology with bird age (pullet or spent hens) as a variable. A total of 144 single comb white leghorns were used for the study. Birds were divided into two ages, pullets coming into lay (<24 weeks) and end of lay cycle (>90 weeks). Both age groups were obtained from the same commercial egg laying facility. Early sexual maturity hens (pullets) were sourced at 16 weeks old and were held for a total of seven weeks. End of sexual maturity hens (spent hens) were sourced at 91 weeks old and were held for a total of nine weeks. Within a given replication, bird age was held constant for all birds in the treatment. Both groups produced eggs at an expected rate while being held prior to depopulation. Birds were housed in a separate facility and moved into the layer hen facility for the trial. All birds in a given trial were instrumented with accelerometers to determine COM. There was a 600 s (10 min) treatment period with a 180 s (3 min) application time, which included during COM data collection. CAFS foam was applied using two foam lines to simultaneously treat both sides of a row of cages. The foam was applied to each cage for a period of 20 s. The foam filled the entire cage in this time period for all of the depopulation trials. During each trial, a total of 15 cages per side with four empty cages, then two cages with four birds each with nine empty cages were treated with foam. Both sides of the cage rows were treated simultaneously, with back-to-back cages being treated. A total of 16 birds were treated per replication. One minute prior to the start of treatment the CAFS system was started and the foam output was observed in order to ensure the desired consistency. All testing was performed under the approval and guidelines of the University of Delaware Institutional Animal Care and Use Committee (Protocol #57R-2016, 18 March 2013) and followed the guidelines laid out by the Federation of Animal Science Societies [19].

### 2.3. General Procedure and Instrumentation

Approximately 24–48 h before a trial, four birds were randomly selected from the flock. Each bird was anesthetized using 5% isoflurane (IsoSol; Vedco, Inc., St. Joseph, MO, USA), was then intubated and placed on 3% isoflurane for maintenance of anesthesia. Three-channel wireless biopotential transmitters (PhysioTel model F50-EEE, Data Sciences, International, St. Paul, MN, USA) were surgically implanted in the back of the neck of each bird. Three leads (two recording leads and one ground lead) were placed on the meninges covering the telencephalon through 0.9 mm holes that were drilled into the parietal bone, two holes on the right side of the midline, and one on the left, using a high speed microdrill (model 18000-17, Fine Science Tools, Foster City, CA, USA). One recording lead was placed on each side of the midline and the ground lead was place on the right side. Two leads were implanted in the complexus muscle just below the skull for electromyography (EMG). All leads were held in place with cyanoacrylate. All birds that underwent surgery received a nonsteroidal anti-inflammatory drug injection (carprofen or meloxicam) for treatment of pain and inflammation. After surgery, the birds were allowed to recover for a period of 24 h. The surgical procedure is based on Savory and Kostal [20,21] and has been used with broilers [8], turkeys [22], layers, and ducks [23,24].

Four RMC-1 PhysioTel (Data Sciences International, St. Paul, MN, USA) receivers were used to record signals from the wireless transmitter. A receiver was placed on the left, right, and backside of the cage of the surgically instrumented bird and the fourth receiver was placed on the top of the cage. Signals from the receivers passed through a Matrix (Data Sciences International). Dataquest A.R.T. acquisition software (Data Sciences International) was used to monitor and record brain activity. The time to unconsciousness and brain death were extracted from the collected data.

EEG files were analyzed in NeuroScore (Data Sciences International) to detect EEG silence (brain death) and unconsciousness. The recorded signal was broken down into four different regions based on an analysis using recorded time as well as EMG and EEG patterns. The raw EEG and EMG signals were analyzed in NeuroScore by adding labeled markers over artifact-free 2-s epochs indicating pretreatment, treatment, terminal convulsions, and post-terminal convulsion periods. The markers were placed based on visual analysis of the EEG signal using the EMG signal as a reference to eliminate motion artifacts. Artifacts appear as a high-amplitude spike in both the EEG and EMG signal. The mean EEG signal, the mean EMG signal, the values for α (8–12 Hz), β (16–24 Hz), δ (0.5–4 Hz), θ (4–8 Hz), and σ (12–16 Hz), the z-ratio, and markers were exported from NeuroScore to Excel (Microsoft Corp., Redmond, WA, USA) and charted. The relative power band ratio of α–δ monitors formed a trend from high frequency brain wave activity to low frequency brain activity with the times to unconsciousness determined by the α / δ ratio [25]. The gross signal was passed through a filter and analyzed for the point of silence (brain death) or where the mean signal over a 1 s period was steady at about 0 µV. 

ATCA was measured using ECG electrodes placed on a previously plucked area on the right wing and both thighs of the surgically instrumented bird. The ECG was calibrated to ensure a normal rhythm and correct placement of leads. The ECG signals were processed through an MP30A acquisition unit (BIOPAC Systems, Inc., Goleta, CA, USA) and were recorded using BIOPAC Student Lab (BSL) software. Analysis of the ECG signals was conducted using BIOPAC Student Lab Pro to determine the onset of ATCA. ATCA was defined as the cessation of rhythmic electrical cardiac activity that consistently results in an isoelectric ECG. The point of ATCA was selected to be after terminal convulsions and associated motion artifacts cease, and arrhythmic electrical activity began [24]. 

An accelerometer (HOBO UA-004, Onset Computer Corporation, Bourne, MA, USA) was attached to the right leg of instrumented birds using a wire tie. Data were collected from the accelerometers using HOBO Data Loggers and were analyzed using Excel [26,27]. For analysis, the X, Y, and Z acceleration channels were vector composited into one channel and the localized flat line was used to determine COM.

### 2.4. Treatment Specifics

A critical difference between the two experiments was the treatment specifications. In Experiment 1, the treatments were CO_2_ gas, a Rowe manufactured CAFS, and a Rowe manufactured CAFS with CO_2_ gas. In Experiment 2, a Hale based CAFS was used. Table 1 summarizes the CAFS specifications for Experiments 1 and 2.

CO_2_ gassing was conducted using a partial house gassing procedure in which a portion of the house was sealed. Sealing was implemented by covering the block of layer cages being treated with thick plastic which was held to the side of the cage with clips forming an airtight container. Final sealing of the chamber was completed just prior to treatment. Carbon dioxide gas was introduced into the chamber at a consistent rate of 510 standard liters per minute (slpm) for the entirety of the 180 s application period. The gas was turned off at the end of the application period and the birds were exposed to CO_2_ for the remainder of the 900 s application period.

The foam was created using a custom compressed air foam system consisting of a mixing chamber for ambient air and CO_2_ that was used to inject a specific amount of CO_2_ gas or air into the foam to keep concentrations consistently at the set amount [17,18]. To produce large enough quantities of CO_2_ gas and to create foam with CO_2_ gas, this system was equipped with a 25 kW generator to supply three-phase power to a Thermax H3L vaporizer (Thermax, Dartmouth, MA, USA). The vaporizer was used to heat liquid CO_2_ to produce the gas being injected into the gas mixing system. For CAFS with ambient air treatments, the air was injected into the system to achieve a 413 kPa outlet pressure. The foam was applied to each cage for a period of 20 s. The foam filled the entire cage in this time period for all of the depopulation trials. One minute prior to the start of treatment, the foam trailer was started and the foam output was observed in order to create the desired consistency. No additional foam was added to make up lost volume due to holes in the cages or bird motion. 

For CAFS with CO_2_, the same equipment was used to create the foam as previously described. However, for this treatment, a CO_2_ evaporator trailer equipped with a manifold was used to inject CO_2_ gas into the foam [17,18]. The CO_2_ gas application rate was 288 slpm at 580 kPa and the air application rate was 1100 slpm at 580 kPa. For both CAFS treatments, the gas mixtures were added to a foam water mixture containing 2% Phoschek WD-881 class A foam. The foam trailer and CO_2_ trailer were started one minute prior to the start of treatment, to allow foam output to be observed in order to create the desired consistency. CAFS with CO_2_ gas was applied to each cage for a period of 20 s. CAFS and CAFS with CO_2_ were directed into the cage and the depth of foam made was assisted by the presence of the manure belt as a barrier.

### 2.5. Data Analysis

The critical time of physiological events was extracted from the EEG, ECG and accelerometers and data were compiled in Excel, and statistical analysis was performed using JMP 13.0 (SAS Institute Inc., Cary, NC, USA). Statistical analysis methods included Fit Y by X analysis using ANOVA, and a Student’s *t*-test of means. All tests were conducted at the 5% (α = 0.05) significance level.

## 3. Results

### 3.1. Experiment 1

All three treatments, CAFS, CAFS with CO_2_, and CO_2_ gas were able to successfully depopulate caged layer hens. Several data sets were removed due to sensor error or signal irregularities, however, sensor data was recorded and analyzed independently and the loss of one sensor output did not result in all data from a given bird being removed.

**Figure 1:**
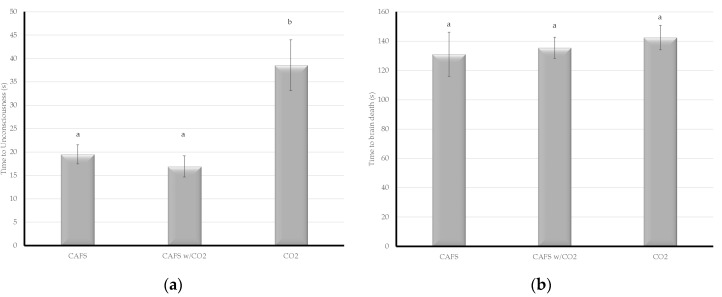
The time from treatment to physiological parameters during depopulation of caged layer hens under one of three treatments was evaluated. Letters within a given chart denote assigned connected letters report with differing letters representing statistical significance. (**a**) Compressed air foam systems (CAFS) and CAFS with CO_2_ resulted in faster unconsciousness; (**b**) there was no statistically significant difference in time to brain death between treatments.

**Figure 2:**
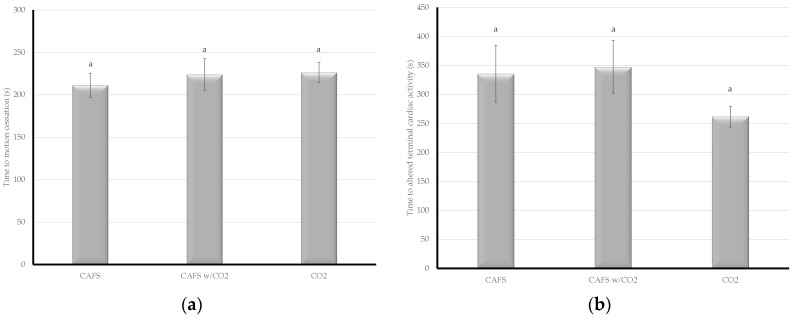
The time from treatment to physiological parameters during depopulation of caged layer hens under one of three treatments was evaluated. Letters within a given chart denote assigned connected letters report with differing letters representing statistical significance. (**a**) There was no statistically significant difference in time to cessation of motion (COM) between treatments; (**b**) there was no statistically significant difference in time to altered terminal cardiac activity between treatments.

The compressed air foam treatments were significantly faster for time to unconsciousness than CO_2_ gas alone (µ = 38.5 s), however there was no significant difference between the foam treatments themselves (CAFS: µ = 19.5 s; CAFS w/CO_2_ gas: µ = 16.9 s) (Figure 1a). Compressed air foam resulted in faster (CAFS: µ = 131.1 s; CAFS w/CO_2_: µ = 135.5 s) times to brain death than CO_2_ gas (µ = 142.4 s), but the difference was not statistically significant (Figure 1b). Compressed air foam was also faster (CAFS: µ = 211.4 s; CAFS w/CO_2_: µ = 224.0 s) to COM than CO_2_ gas (µ = 226.4 s), but again the results were not significant (Figure 2a). Carbon dioxide gas was faster (µ = 261.9 s) to ATCA than both of the foam treatments (CAFS: µ = 335.7 s; CAFS w/CO_2_ gas: µ = 347.4 s), although the results were not significant (Figure 2b). 

### 3.2. Experiment 2

The second CAFS was also able to depopulate caged birds. Lethality improved with successive trials as the process was improved from 62% in trial 1 to 100% in the later trials. 

**Figure 3:**
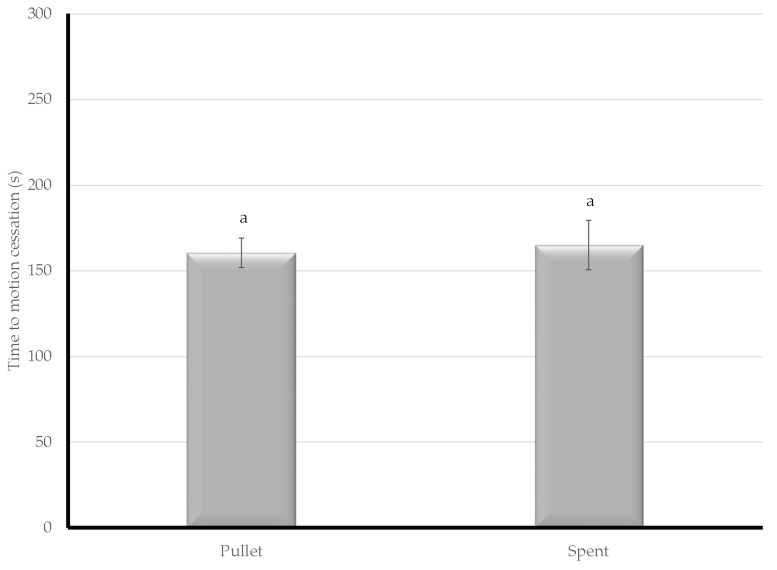
The time from treatment to cessation of movement was evaluated using a second CAFS system with birds of differing ages. Letters denote assigned connected letters report with differing letters representing statistical significance.

Bird age was a variable in Experiment 2 and pullets had a faster time to COM than spent hens (pullet µ = 161.9 s; spent µ = 164.2 s), however, the results were not significant (Figure 3).

### 3.3. Experiment 1 versus Experiment 2

**Figure 4:**
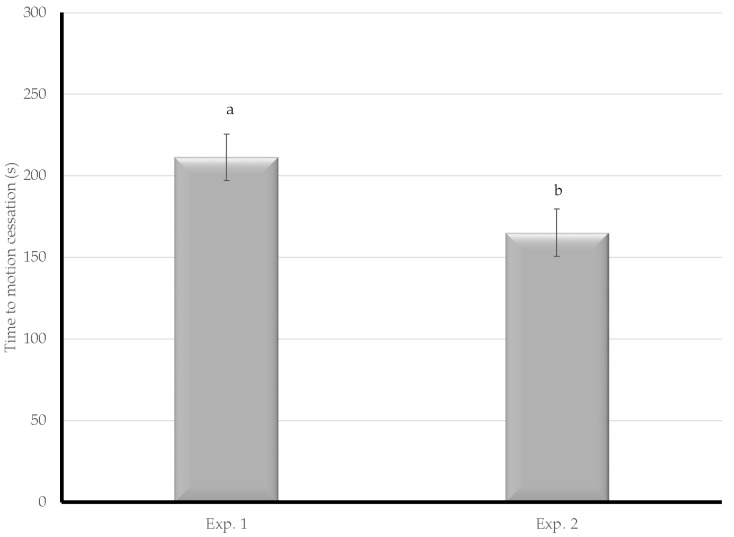
The time from treatment to cessation of movement of spent hens was compared between Experiments 1 and 2. Letters denote assigned connected letters report with differing letters representing statistical significance.

The time to COM of the two procedures was similar, in both experiments. The difference between the two treatments was significant with the Hale CAFS in Experiment 2 (µ = 163.0 s) being faster than the Rowe CAFS in Experiment 1 (µ = 220.4 s) as shown in Figure 4.

## 4. Discussion

Previous research documented that water-based foam or CO_2_ gas could be used to depopulate layer hens outside of a cage [28]. Two different types of CAFS, with and without CO_2_, were able to depopulate caged layer hens, something that other types of foam depopulation were not able to achieve. The results of this project complemented Gurung et al. [17,18], who documented the effectiveness of CAFS, but did not assess unconsciousness. 

The two experiments reported here, in connection with Gurung et al. [17,18], illustrate that the mean differences in time to COM depend on implementation (Experiment 1 = 211 s, Experiment 2 = 165 s, Gurung et al. = 195 s). The equipment used in Experiment 1 and that used by Grunung et al. [17] was similar; however, there were differences in implementation. In Experiment 2, foam was applied from both sides of the cage simultaneously, which may result in a more rapid cage fill time. Multiple CAFS discharge lines, however, are more difficult to flow balance without surging. The CAFS evaluated systems are suitable for research but will need additional development to create a field ready prototype suitable for large scale commercial poultry facilities. 

The effectiveness of the system in Experiment 1 was sufficient, but was initially insufficient for Experiment 2. There are few viable alternatives to using animal models for developing depopulation or euthanasia procedures, which presents a significant challenge. This was seen dramatically in Experiment 2, where initial treatments did not have viable lethality for a depopulation procedure. By the end of Experiment 2, lethality reached 100% with changes in how foam would be applied into the cage.

Existing foam generators and air aspirating nozzle foam depopulation systems are intended to provide medium–high expansion foam suitable for depopulating floor-reared poultry. For foam depopulation systems intended for floor-reared poultry, foam tends to either be blocked from penetrating the cage walls or passes through the floor with insufficient residency time. For caged birds, wire cage walls serve as a nucleation site for the foam, and foams with higher expansion ratios tend to strike the cage and the majority of the foam does not penetrate the cage. With lower expansion foams, the foam passes through the cage wall, but also passes too quickly through the cage floor, leaving insufficient quantity of foam inside the cage for depopulation. The foam characteristics of CAFS can be changed by altering foam concentrate, foam concentrate concentration, air injection rate, and water flow rate. By changing the CAFS foam characteristics, we achieved a foam with the correct balance of penetration and retention to depopulate birds in a cage with a manure belt. 

CAFS, CAFS with CO_2_, and CO_2_ gas resulted in similar times for all characteristics observed, except unconsciousness. From a welfare point of view, the time it takes to reach unconsciousness is the most significant because it shows the point at which the bird is no longer able to feel pain and would not be aware of their surroundings. Unconsciousness is the first physiological characteristic observed. This is followed by brain death, which is followed by terminal convulsions. COM assesses the end of terminal convulsions and immediately follows brain death. Experiment 2, which uses only COM, represents the period of time post unconsciousness and brain death, therefore, is beyond the point at which a bird would be potentially conscious and aware.

CAFS inherently includes a type of gas, normally compressed air. Of the foam depopulation systems available, dry foam [15,29] and CAFS [17,18] can more readily include alternate gases that would not be practical with air aspirating nozzles or foam generators. Most alternative gases suitable for CAFS depopulation significantly increase the logistical requirements to vaporize and deliver large quantities of gas. Carbon dioxide has been used extensively as a depopulating agent, and could result in improved bird welfare or impact birds not directly reached with foam depopulation. The results of this project show minimal advantage to incorporating CO_2_ in CAFS. Carbon dioxide may have adverse impacts on foam quality and expansion rate [17,18]. Nitrogen serves as an oxygen displacer and is a potential alternative CAFS gas that may have advantages for depopulation without compromising foam quality [17,18]. Gurung et al. [17,18], noted that CAFS with N_2_ resulted in a faster time to motion cessation than CAFS or CAFS with CO_2_. 

CAFS and air aspirating nozzles can be used to depopulate layer hens, however, the air aspirating nozzles are only suitable for birds not confined to a cage. When comparing the results of this study to previous research [28], CAFS and CAFS with CO_2_ resulted in a faster time to unconsciousness than with an air aspirating nozzle (CAFS = 19 s, CAFS w/CO_2_ = 17 s, nozzle = 41 s). Compressed air foams also resulted in faster times to brain death than an air aspirating nozzle (CAFS = 131 s; CAFS w/CO_2_ = 135 s, nozzle = 183 s). The air aspirating nozzle resulted in materially faster times to terminal convulsions and cessation of motion than CAFS or CAFS w/CO_2_ (CAFS = 211 s; CAFS w/CO_2_ = 224 s, nozzle = 147 s). Similarly, the nozzle resulted in faster ATCA than either CAFS treatments (CAFS = 336 s; CAFS w/CO_2_ = 347 s, nozzle = 177 s). The experiments use similar analysis metrics, but different protocols, with the air aspirating nozzle experiments using a single bird at a time in a container, and the CAFS treatments including multiple birds in separate cages during the same foam application. This is an important distinction, since there is a ‘per cage’ or ‘per bird’ treatment time for multiple bird trials that cannot be readily accounted for.

All depopulation procedures for a large egg laying complex require removing birds from their cages either pre-treatment or post-mortem; there are few available depopulation alternatives for a large egg laying complex. Removal of live, infected, or potentially infected birds places human responders at risk. Removal of contaminated carcasses also places human responders at risk, and requires handling decomposing carcasses during removal. Whole-house CO_2_ gassing is one alternative depopulation method that has been successfully used for caged layers. This method, like the CAFS method, has the advantage of limited handling of the birds prior to depopulation and can be accomplished much quicker than the containerized method. However, whole-house CO_2_ gassing can only be effective in a house that can be tightly sealed; this requires the use of large quantities of carbon dioxide that causes significant drops in temperatures inside the house during the process. Sealing can be difficult and time consuming if the facility is large or has experienced any structural damage. Partial-house gassing may be difficult to implement in a facility with fixed cages. Containerized gassing provides good control of gas concentration when carts are properly air tight, but is very time and labor intensive. Additionally, CO_2_ gassing poses a welfare threat to humans. Responders performing whole-house gassing must wear a self-contained breathing apparatus and appropriate gas concentration meters when entering a treated house. 

## 5. Conclusions

During disease outbreaks, depopulation of large egg laying facilities poses a particular challenge, since their cage arrangements make it difficult to successfully reach and treat the birds. Water-based foam has been successfully used for floor-reared poultry, but not for caged birds. In this experiment, a different type of foam depopulation equipment (compressed air foam or CAFS) was successfully used to depopulate caged layer hens. CAFS, with or without CO_2_, resulted in faster unconsciousness than CO_2_ gas, however, time to brain death, motion cessation, and altered terminal cardiac activity was not statistically different. CAFS with CO_2_ did not provide sufficient advantages over CAFS to balance the increased logistical requirements.

## Figures and Tables

**Table 1:** Summary of the compressed air foam systems (CAFS) for Experiments 1 and 2.

Item	Experiment 1	Experiment 2
Pump	567 L per minute (Hale Products, Inc., Ocala, FL, USA)	Hale 240 GPX (Hale Products, Inc., Ocala, FL, USA)
Prime Mover	Kohler 30 kW (Kohler, WI USA)	Briggs and Stratton 26 kW (Wauwatosa, WI, USA)
Foam Proportioner	FoamPro (Kingston, NY, USA)	Hale Foam Logics 2.1A (Ocala, FL, USA)
Foam Concentrate	Phos-Chek WD-881 (ICL Performance Products, LLC, St. Louis, MO, USA)	
Discharge	3.8 cm initial, 6.5 cm smooth bore discharge	4.4 cm initial, 7.6 cm smooth bore discharge
